# Different Flowering Strategies Ensure Reproductive Success in Two Coexisting Self‐Incompatible Orchids

**DOI:** 10.1002/ece3.72843

**Published:** 2026-01-02

**Authors:** Shi‐Mao Wu, Sheng Zhang, Yi‐Hua Wu, Xiang‐Gui Chen, Jiang‐Yun Gao

**Affiliations:** ^1^ State Key Laboratory of Vegetation Structure, Function and Construction (VegLab), Institute of Biodiversity, School of Ecology and Environmental Science Yunnan University Kunming China; ^2^ Yunnan Key Laboratory for Integrative Conservation of Plant Species With Extremely Small Populations, Kunming Institute of Botany Chinese Academy of Sciences Kunming China; ^3^ Yunnan Forestry Technological College Kunming China

**Keywords:** *Coelogyne*, flowering strategies, geitonogamy, *Pholidota*, reproductive success, self‐incompatibility

## Abstract

Orchids have evolved diverse reproductive strategies to overcome pollinator limitation and pollen discounting from geitonogamy, particularly in self‐incompatible species. This study compares two coexisting, self‐incompatible orchids (*Pholidota articulata* and *Coelogyne prolifera*) sharing pollinators in an ancient tea garden, examining how their contrasting flowering strategies enhance reproductive success. We conducted a 3‐year study analyzing flowering phenology, floral traits, pollinator behavior, pollinia removal and deposition, the breeding system, and fruit set under both natural conditions and from hand‐pollination treatments. Despite partial flowering overlap, the species exhibited distinct strategies: *P. articulata* employed a mass‐flowering strategy with large floral displays, high nectar rewards, and synchronized anthesis (all flowers per inflorescence opening within ~4 days), while *C. prolifera* adopted a steady‐state strategy with prolonged single‐flower longevity (~13 days) and extended flowering duration (60 days) and consistent but comparatively lower nectar secretion. Both species shared two pollinator species (*Vespa velutina* and *Vespa mandarinia*) in 3 years, but the visit frequency was consistently higher for *P. articulata* . Remarkably, 83.3% ± 6.5% of *P. articulata* flowers were pollinated on their first day versus only 4.5% ± 8.6% in *C. prolifera* . Although pollinia removal and deposition peaked during initial anthesis in *P. articulata* , *C. prolifera* showed lower pollen discounting (44.4%) throughout flowering. Despite these differences, both maintained moderate natural fruit sets (21.2%–30.7%) across years, which are substantially higher than the typical 2% reported for most self‐incompatible orchids. Our findings demonstrate that coexisting, self‐incompatible orchids sharing pollinators can achieve comparable reproductive success through divergent strategies: *P. articulata* maximizes pollination efficiency via synchronized mass‐flowering, while *C. prolifera* enhances pollination opportunities through prolonged flowering. This highlights the adaptive diversity of flowering strategies in self‐incompatible orchids facing pollinator limitation and geitonogamy.

## Graphical abstract

This study compares the reproductive strategies of two self‐incompatible orchids, *Pholidota articulata* (mass‐flowering) and *Coelogyne prolifera* (steady‐state flowering). Despite differences in pollinator visitation and pollinia transfer efficiency—higher in *P. articulata* —both species achieved similar natural fruit set rates over 3 years. The findings suggest that distinct flowering strategies can equally ensure reproductive success in self‐incompatible orchids.

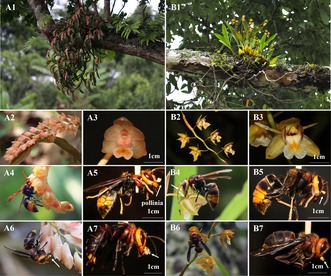

## 1 Introduction

Pollination limitation represents a fundamental constraint in orchid reproductive ecology, exerting significant selective pressures on the evolution of this diverse plant family (Tremblay et al. 2005). The Orchidaceae has responded to this constraint through remarkable evolutionary innovations, including extraordinary species diversification, specialized floral traits, and complex pollination strategies (Dressler 2005; Darwin 1862; Jersáková et al. 2006; Wu and Gao 2024). As predominantly outcrossing species that rely exclusively on animal pollinators for sexual reproduction (Swarts and Dixon 2009), orchids typically exhibit specialized pollination systems (Gill 1989; Burd 1994; Wilson et al. 1994; Sun et al. 2006). Consequently, orchid fruit sets are generally low, often falling below 10% in nonautogamous species (Janzen et al. 1980; Schemske 1980; Nilsson et al. 1986; Ackerman 1989; Zimmerman and Aide 1989; Calvo 1990a; Christensen 1992), with pollinator limitation and self‐incompatibility identified as primary constraints rather than resource limitation (Calvo 1993; Tremblay et al. 2005). Moreover, even in rewarding and self‐incompatible species, substantial pollen loss due to geitonogamy can lead to low fruit set despite high pollinaria removal and deposition rates (Castro et al. 2022).

Flowering plants exhibit substantial variation in reproductive phenology, with two predominant strategies emerging along a continuum: mass‐flowering (characterized by synchronous production of numerous flowers over brief periods) and steady‐state flowering (featuring asynchronous production of few flowers over extended durations) (Gentry 1974). These contrasting strategies fundamentally influence plant‐pollinator interactions and reproductive outcomes (Rathcke and Lacey 1985), with steady‐state flowering generally associated with higher outcrossing rates (Augspurger 1980). In rewarding species, nectar serves as the primary pollinator attractant, increasing visitation frequency (Schmid‐Hempel 1987) and typically resulting in higher fruit sets compared to deceptive species (Tremblay et al. 2005). However, both strategies face pollinator limitation challenges (Ackerman and Montalvo 1990; Calvo 1990b; Nilsson 1992; Fritz and Nilsson 1994; Neiland and Wilcock 1998), with mass‐flowering potentially increasing geitonogamy and subsequent pollen discounting (Holsinger et al. 1984). This creates an evolutionary trade‐off between pollinator attraction and mating quality (Harder et al. 2004), particularly pronounced in self‐compatible species where high fruit sets may correlate with reduced seed viability (Zhang and Gao 2021).

Angiosperms have evolved numerous mechanisms to optimize pollination efficiency while minimizing self‐pollination, with self‐incompatibility representing the most effective genetic barrier to inbreeding (Richards 1997). This system maintains genetic diversity in plant populations (Hamrick and Godt 1990; Borba, Félix, et al. 2001). Many orchid species are self‐compatible and employ various prepollination mechanisms to prevent autogamy (Dressler 1993; Borba and Semir 1999, 2001). In other species that are self‐incompatible, geitonogamy persists as a significant reproductive constraint, causing ovule and pollen discounting (Liu et al. 2013) and frequently resulting in floral abortion (Tremblay et al. 2005). Various antigeitonogamy mechanisms have been documented, including sequential flowering (Ackerman 1989; Aragón and Ackerman 2001), dichogamy/protandry (Ackerman 1975; Catling 1983; Singer and Sazima 2001a, 2001b; Singer 2002; Singer and Koehler 2002), pollinarium movement (Darwin 1877; Castro et al. 2022), and specialized pollination structures (Liu et al. 2013).

During our investigation of orchid diversity in the ancient tea gardens of southwest Yunnan, we identified two coexisting species (*Pholidota articulata* and *Coelogyne prolifera*), which frequently grow on the same shade trees and exhibit partially overlapping flowering periods in the LaoBanZhang ancient tea garden (LBZ). These ecologically dominant species (with 284 and 313 flowering individuals, respectively) represent ideal models for studying reproductive strategies in self‐incompatible orchids (Wu et al. 2024). Given the extreme reproductive constraints typically faced by self‐incompatible orchids (often < 2% fruit set; Tremblay et al. 2005), this study aims to: (1) characterize differences in flowering patterns and floral traits between these species; (2) identify pollinator assemblages and visitation patterns; and (3) elucidate mechanisms by which these ecologically important but understudied species reduce geitonogamy while maintaining reproductive success.

## 2 Materials and Methods

### 2.1 Study Species and Site Description

The epiphytic orchids *P. articulata* and *C. prolifera* (Orchidaceae) are widely distributed across tropical and subtropical Asia. Representing two speciose genera (*Pholidota*: ~30 species; *Coelogyne*: ~200 species), these taxa exhibit distinct biogeographic patterns in China (12 and 31 species, respectively), with 5 and 12 species recorded in Xishuangbanna (Chen and Coelogyne 2009; Gao et al. 2014). Both species display racemose inflorescences and synchronous May flowering, yet differ morphologically: *P. articulata* produces greenish‐white flowers at pseudobulb apices, while *C. prolifera* bears yellowish‐green flowers emerging from pseudobulb‐leaf junctions. Notably, May marks the beginning of the rainy season in this region (Gao et al. 2014).

**FIGURE 1:**
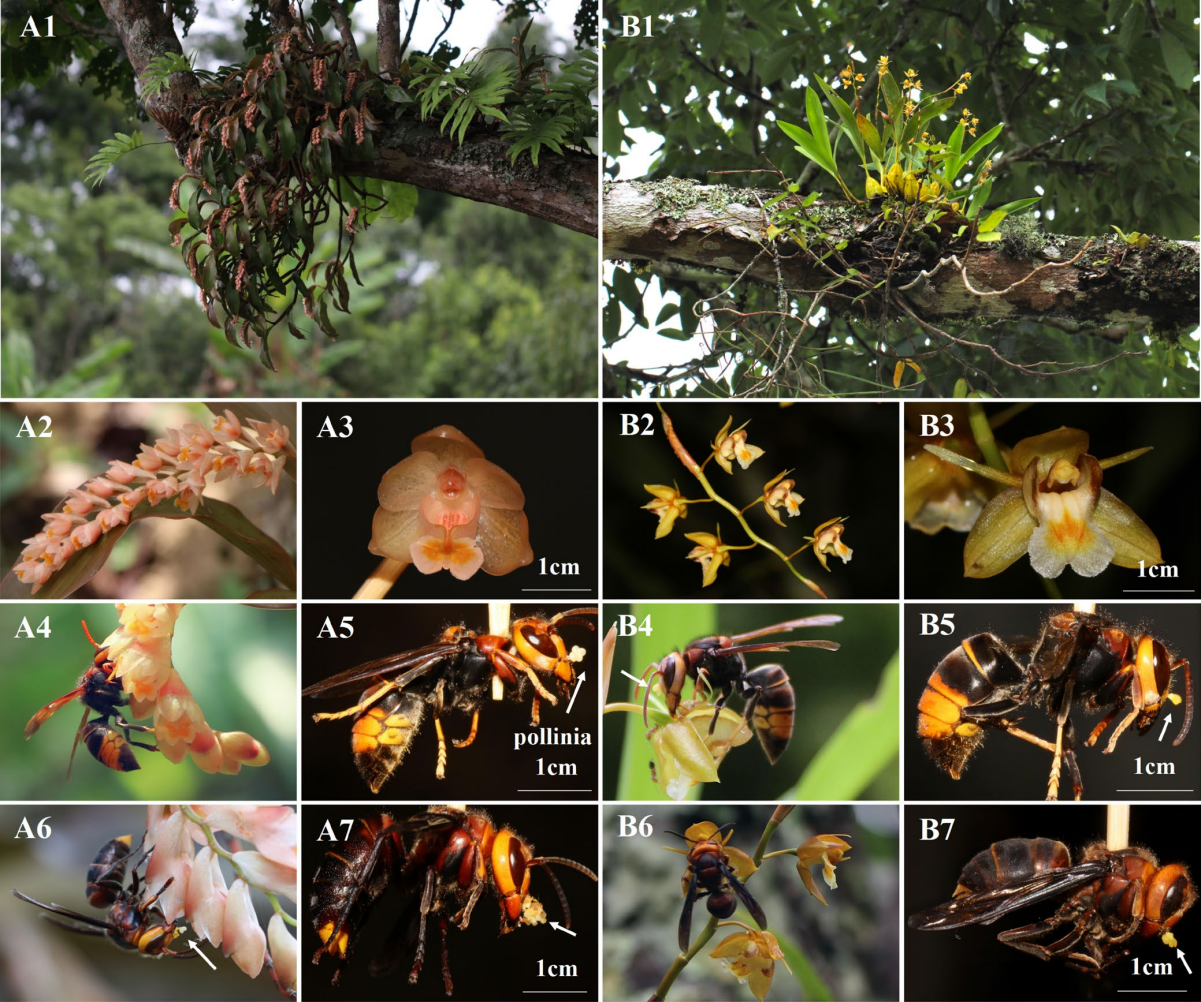
Plant, inflorescences, flowers, and pollinators of *P. articulata* and *C. prolifera*. Plant and habitat of *P. articulata* (A1) *and C. prolifera* (B1). Inflorescence and single flower of *P. articulata* (A2, A3) and *C. prolifera* (B2, B3). *Vespa velutina* and *V. mandarinia* visit the flowers of *P. articulata* (A4, A6) and *C. prolifera* (B4, B6) with pollinia attached to their heads (A5, A7, B5, B7), respectively.

Fieldwork was conducted in the LBZ (21°43′41″ N, 100°30′23″ E; 1700–1900 m asl) in Xishuangbanna, Yunnan Province, China. This subtropical montane ecosystem experiences mean annual temperatures of 18.7°C and precipitation of 1342–1540 mm. The study species exhibited co‐occurring distribution on ancient tea trees and shade trees (Figure 1A1,B1), with *P. articulata* demonstrating a broader elevational range (800–2500 m) than *C. prolifera* (1200–2000 m) (Gao et al. 2014). Data collection spanned the 2019–2022 flowering and fruiting seasons.

### 2.2 Phenological and Floral Trait Analysis

To compare the flowering phenological traits between *P. articulata* and *C. prolifera* , we randomly selected and observed at least 70 individual flowering plants of each species during the 2021 flowering season. The observations were recorded for further analysis. The number of flowering inflorescences, the number of flowers on each inflorescence, the sequential order of open flowers, and the timing of flowering were observed and counted daily. The floral longevity of emasculated (pollinia‐removed), pollinated, and intact flowers was compared by marking 20 flowers from five individuals for each treatment. We selected different individuals for each treatment. We judge a flower as “opening” or “wilting” based on whether it has the role of attracting pollinators in the pollination process. For example, a flower was judged as “opening” when the labellum was spread and as “wilting” when its color or shape changed. The daily proportion of flowering on each marked plant was recorded, along with the times of opening and wilting, in order to determine the degree of overlap in the flowering period for both species.

We studied the floral morphology during the flowering period in 2022. We removed the entire pollinarium from newly open flowers of both species and observed, within a 0–5 min timeframe, whether the caudicle or stipe exhibited any movement. Subsequently, we monitored changes in the stigma cavity over several hours following pollination. The 20 newly opened flowers from 10 different individuals of each species were randomly selected to measure the flower size using an electronic vernier caliper. Additionally, we randomly selected 20 inflorescences from 20 individuals that were bagged before anthesis to measure the volume of nectar and the concentration of sucrose in the nectar. To measure the volume of nectar per flower, we randomly selected 40 newly opened flowers from 10 individuals and used 10‐μl Sigma “microcap” calibrated capillary tubes (Sigma Chemical Co., St. Louis, USA) between 12:00 and 14:00. To measure the total sugars concentration in the nectar, we prepared 10 samples, each containing a mixture of 10 flowers from an individual (the volume of nectar in a single flower does not meet the measurement standard). We used a handheld, temperature‐compensated refractometer (Eclipse, Bellingham and Stanley Ltd., UK) simultaneously for each species.

### 2.3 Pollinator Observations

The pollinator observations for each species were made during the flowering seasons for three consecutive years (2019–2021) in the LBZ ancient tea garden. Observations for the two species were mainly conducted from 8:00 to 19:00 local time each day. For each observation, 10 inflorescences from one individual were randomly selected. Individual pollinators visiting the two orchids were carefully observed to determine their pollination behavior. This included recording the average visiting times per hour and the number of flowers visited per inflorescence. In addition, we compared the differences in pollinator visitation frequency to the two orchid species between sunny and rainy days, with 20 h of observation conducted for each condition. All species of visitors were photographed during the observation periods. To identify the species and observe the deposition of pollinia on different parts of their bodies, we attempted to capture 5–10 individuals of the pollinators per species at the end of the observation period. Vouchers for both the orchid species and pollinators examined in this study have been deposited in the Herbarium and Collection of the School of Ecology and Environmental Science, Yunnan University.

### 2.4 Hand Pollination Experiments

To investigate the breeding systems of *P. articulata* and *C. prolifera* , three hand‐pollination treatments were conducted in situ during the 2022 flowering season: (1) Bagging: a total of 150 flowers (from 20 inflorescences) of *P. articulata* and 50 flowers (from 20 inflorescences) of *C. prolifera* were enclosed in mesh bags to exclude pollinators. (2) Self‐pollination: prior to anthesis, 95 flowers (from 20 inflorescences) of *P. articulata* and 100 flowers (from 30 inflorescences) of *C. prolifera* were bagged and subsequently hand‐pollinated using pollen from either the same flower or another flower on the same individual. (3) Cross‐pollination: before flower opening, 305 flowers (from 15 inflorescences) of *P. articulata* and 48 flowers (from 10 inflorescences) of *C. prolifera* were bagged and manually pollinated with pollen sourced from conspecific individuals located at least 50 m away. Fruit set was assessed for each treatment following the conclusion of the flowering period.

To test for potential interspecific reproductive barriers in the absence of natural hybrids, we performed reciprocal cross‐pollinations between *P. articulata* and *C. prolifera* following standard methods (Dafni 1992). A total of 100 flowers (50 per direction) from 30 inflorescences were used, and fruit set was assessed at the end of the reproductive season in 2022.

### 2.5 Pollinia Removal, Deposition, and Fruit‐Sets

To estimate male and female reproductive success, we marked 31 inflorescences of *P. articulata* and 36 inflorescences of *C. prolifera* (from a total of 30 individuals) before anthesis in 2021. We monitored and recorded the removal and deposition of pollinia on a daily basis, starting from the first day of flowers. After anthesis (whole flowering period), the proportion of pollinia removal, deposition, and fruit set during the entire flowering period was calculated by dividing the number of flowers with pollinia removed, deposited, and fruits produced by the total number of flowers examined.

The natural fruit sets of two species were investigated for three consecutive years (from 2019 to 2021) by marking a larger number of individuals. The numbers of flowers, inflorescences, and individuals that were used are summarized in Table 2.

### 2.6 Data Analysis

Differences in all phenology traits, floral traits, the number of flowers visited per inflorescence by different pollinators, and average natural fruit sets over 3 years were analyzed using a one‐way ANOVA. A General Linear Model (GLM) was employed to analyze the variation in pollinator visitation rates (visits per hour) across different years and between orchid species. The pollinia removal, deposition, fruit set of hand cross‐pollination, and natural fruit set were compared using Mann–Whitney tests. All statistical analyses were performed by R (version 4.4.2).

## 3 Results

### 3.1 Phenology and Floral Traits

**FIGURE 2:**
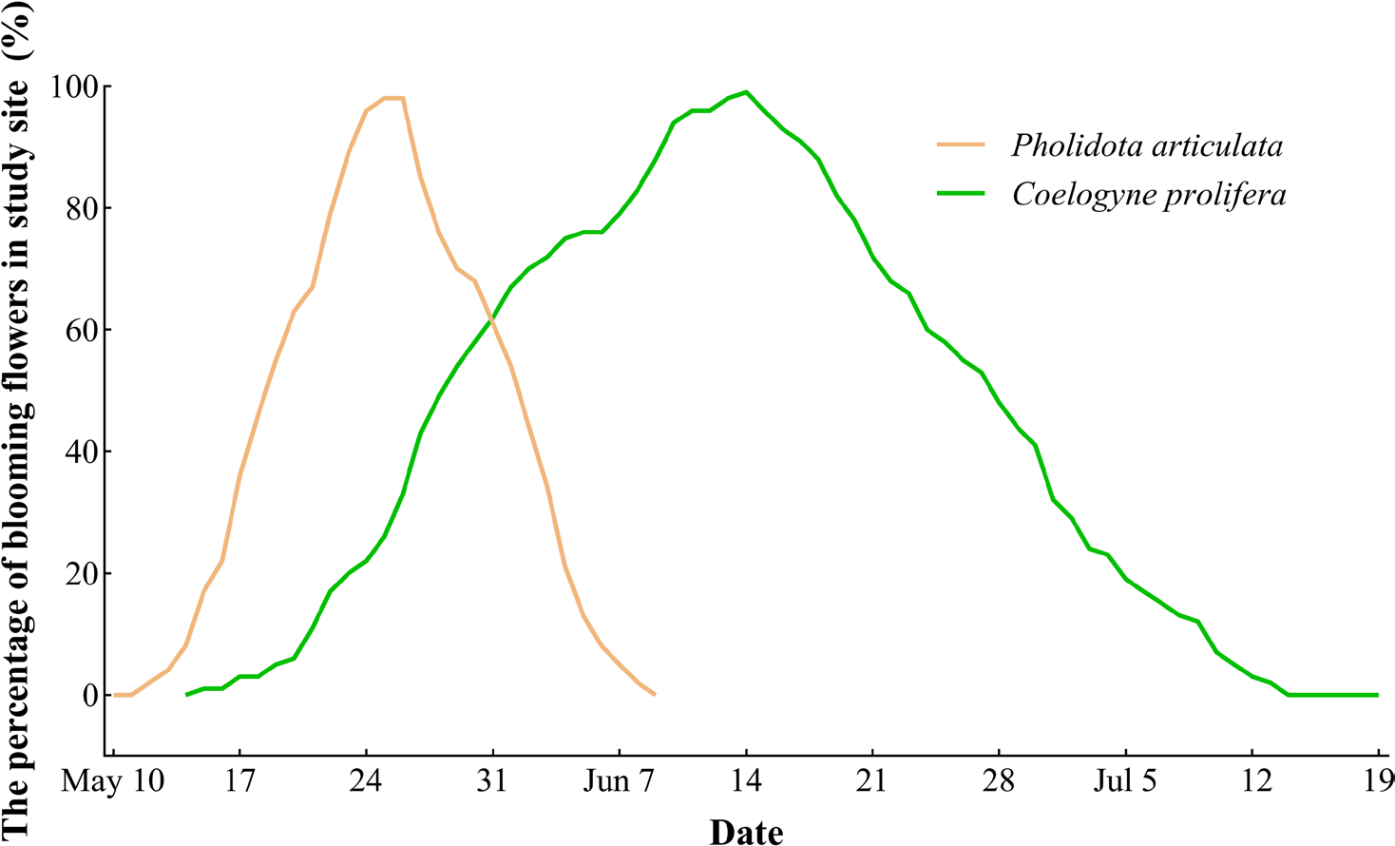
Flowering phenology (the percentage of blooming flowers per day) of *P. articulata* and *C. prolifera* in our study site.

The two orchids exhibited flowering activity between May and July, coinciding with the onset of the rainy season (May–October) at our Xishuangbanna study site. Our 2021 observations revealed partial overlap in their flowering phenologies (Figure 2), with *P. articulata* flowering from May 12 to June 9 (28 days duration) and *C. prolifera* from May 15 to July 13 (60 days duration) (Table 1). Floral longevity differed significantly between species: in *P. articulata* , intact flowers lasted 4.65 ± 0.73 days (mean ± SD; *n* = 20), significantly longer than emasculated (2.55 ± 0.59 days; *p* < 0.001) or pollinated flowers (2.40 ± 0.49 days; *p* < 0.001). For *C. prolifera* , intact flowers persisted for 13.05 ± 1.36 days, showing no significant difference from emasculated flowers (12.40 ± 0.48 days; *p* = 0.12) but significantly longer than pollinated flowers (7.50 ± 0.81 days; *p* < 0.001). Notably, *C. prolifera* flowers exhibited significantly greater longevity than *P. articulata* (*p* < 0.001; Table 1).

**TABLE 1:** Floral traits of *Pholidota articulata* and *Coelogyne prolifera* (mean ± SD) in our study.

Floral traits	*P. articulata*	*C. prolifera*	*F*	*p*
Flower length (mm)	17.78 ± 0.70 (*n* = 20)	18.25 ± 0.78 (*n* = 20)	3.762	*p* = 0.060
Flower width (mm)	10.89 ± 0.75 (*n* = 20)	11.32 ± 0.71 (*n* = 20)	3.225	*p* = 0.080
Nectar volume (μL)	4.36 ± 2.91 (*n* = 40) (0.6–11)	2.62 ± 0.76 (*n* = 40) (0.8–4.2)	13.041	*p* < 0.001
Nectar sugar concentration (%)	29.35% ± 2.67% (*n* = 10) (25.8–34.0)	23.93% ± 3.27% (*n* = 10) (20.0–29.8)	14.836	*p* < 0.001
Flowers per inflorescence	18.75 ± 6.29 (*n* = 36)	4.61 ± 0.68 flowers (*n* = 36)	175.004	*p* < 0.001
Inflorescence per individual	21.07 ± 9.70 (*n* = 30)	6.3 ± 3.18 (*n* = 30)	60.723	*p* < 0.001
Floral longevity (days)	4.65 ± 0.73 (*n* = 20)	13.05 ± 1.36 (*n* = 20)	564.480	*p* < 0.001
The time of all flowers opening per inflorescence (days)	4.29 ± 0.71 (*n* = 34)	10.32 ± 1.51 (*n* = 34)	431.629	*p* < 0.001
Flowering periods (days)	28	60	—	*—*

*Note:* Statistically homogeneous groupings based on a one‐way ANOVA.

**FIGURE 3:**
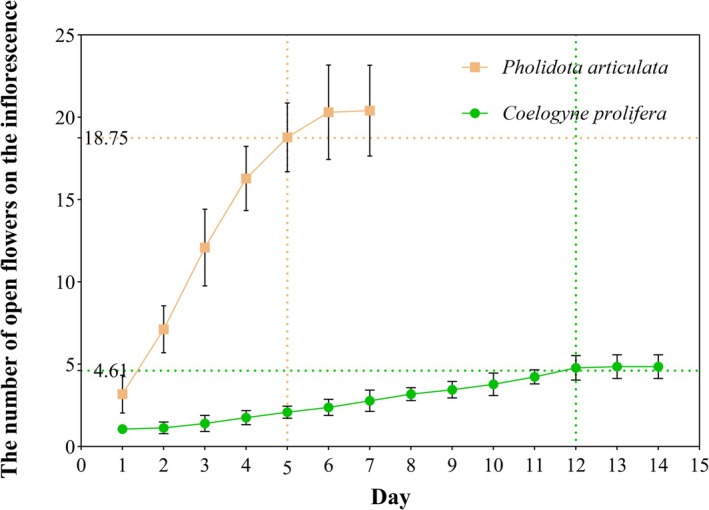
Flowering progression on inflorescences of *P. articulata* and *C. prolifera* in 2021. The horizontal dashed line indicates the mean flower number per inflorescence for each species (18.75 ± 6.29 for *P. articulata* ; 4.61 ± 0.68 for *C. prolifera* ). Vertical dashed lines mark the days when open flower counts reached these mean values (day 5 for *P. articulata* ; day 12 for *C. prolifera* ).

Both species displayed sequential flowering patterns but differed in inflorescence architecture. *P. articulata* produced tightly arranged inflorescences (21.07 ± 9.70 inflorescences/plant; *n* = 30) with flowers opening centripetally (18.75 ± 6.29 flowers/inflorescence; *n* = 36; Figure 1A2), reaching peak flowering by day 5 (Figure 3). In contrast, *C. prolifera* formed loose inflorescences (6.30 ± 3.18 inflorescences/plant; *n* = 30) with basipetal flowering (4.61 ± 0.68 flowers/inflorescence; *n* = 36; Figure 1B2), attaining maximum floral display by day 12 (Figure 3). The floral display (number of flowers and inflorescences) of *P. articulata* significantly exceeded that of *C. prolifera* (all *p* < 0.001; Table 1).

Floral morphology showed convergence between species, with both possessing four entire and indivisible pollinia per flower, each bearing a well‐defined viscidium and lacking nectar spurs. Neither species exhibited caudicle or stipe movement within 5 min of pollinarium removal. Following pollination, the rostellum covered the stigma cavity within 3–4 h, preventing additional pollen deposition. While flower dimensions were statistically similar between species (length: *p* = 0.06; width: *p* = 0.08; Figure 1A3,B3; Table 1), nectar rewards differed substantially. Both species secreted nectar directly onto the labellum, but *P. articulata* produced significantly more nectar with a higher sugar concentration than *C. prolifera* (all *p* < 0.001; Table 1).

### 3.2 Pollinator Observations

Pollinator observations totaled 200 h for *P. articulata* and 210 h for *C. prolifera* from 2019 to 2021. We recorded five insect species visiting *P. articulata* and four visiting *C. prolifera* , but only two wasp species—*V. velutina* and *V. mandarinia*—were confirmed as shared primary pollinators through direct observation of pollinaria attachment (Figure 1A4,A6,B4,B6). The pollinaria of both orchid species were consistently deposited on similar head regions of these wasps (Figure 1A5,A7,B5,B7). *Bombus breviceps* , despite showing the highest visitation frequency to *C. prolifera* flowers (2019: 7.23 ± 2.80; 2020: 7.93 ± 3.29; 2021: 7.43 ± 3.23 visits/h; *n* = 20 h annually), was identified as a nectar robber as no pollinaria were found on its body during the 3‐year study.

**FIGURE 4:**
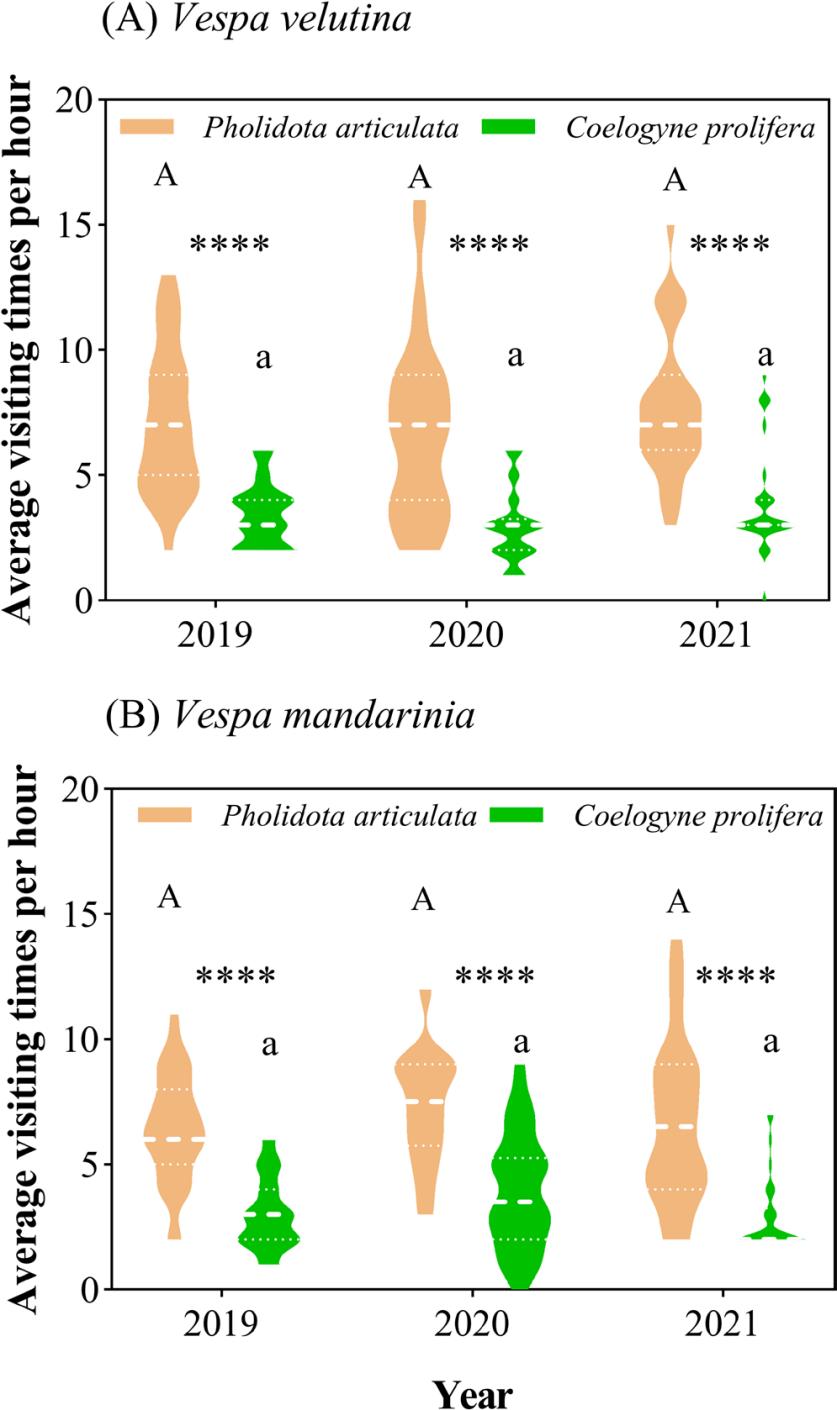
Average visiting times per hour of shared pollinators of *P. articulata* and *C. prolifera* in different years. (A) *V. velutina* ; (B) *V. mandarinia*. Asterisks indicate levels of statistical significance (****p < 0.0001). Statistically homogeneous groupings are marked by the same letter (a/A).

The two shared pollinator species exhibited consistent visitation patterns to both orchids. During peak visitation periods (30 observation hours/species/year), *V. velutina* visited *P. articulata* 214, 202, and 232 times in 2019–2021, respectively, compared to 103, 89, and 116 visits to *C. prolifera* . Similarly, *V. mandarinia* visited *P. articulata* 193, 218, and 199 times versus 91, 118, and 87 visits to *C. prolifera* . While neither wasp species showed significant interannual variation in visitation frequency to either orchid ( *P. articulata* : *P* _*V. velutina*_ = 0.3726, *P*_*V. mandarinia*_ = 0.3423; *C. prolifera* : *P* _*V. velutina*_ = 0.1109, *P*_*V. mandarinia*_ = 0.1193; Figure 4), both species visited *P. articulata* significantly more frequently than *C. prolifera* across all years (all *p* < 0.0001; Figure 4). In addition, pollinator visitation frequency was significantly lower on rainy days (*P. articulata*, 2.65 ± 1.27; *C. prolifera*, 2.10 ± 1.48; *n* = 20) than on sunny days (*P. articulata*, 10.60 ± 3.07; *C. prolifera*, 7.95 ± 1.88; *n* = 20; *p* < 0.0001) for both species.

At the inflorescence level, the two wasp species showed similar visitation patterns within each orchid species. For *P. articulata* , *V. velutina* visited 4.23 ± 1.20 flowers per inflorescence (*n* = 22) compared to 4.58 ± 1.75 for *V. mandarinia* (*n* = 24; *p* = 0.4405, *t* = 0.7783). In *C. prolifera* , visits per inflorescence were 2.73 ± 1.05 ( *V. velutina* , *n* = 22) and 2.43 ± 0.88 (*V. mandarinia*, *n* = 23; *p* = 0.3266, *t* = 0.9922). However, both wasp species visited significantly more flowers per inflorescence in *P. articulata* than in *C. prolifera* ( *V. velutina* : *p* < 0.0001, *t* = 4.300; *V. mandarinia*: *p* < 0.0001, *t* = 5.164).

### 3.3 Hand Pollination Experiments

Both orchid species exhibited complete self‐incompatibility, with all bagged (intact flowers) and hand self‐pollinated flowers failing to produce fruit. Hand cross‐pollination treatments revealed similarly high fruit set rates between species ( *P. articulata* : 87.83% ± 6.94%, *n* = 15 inflorescences; *C. prolifera* : 90.17% ± 10.07%, *n* = 10 inflorescences; *p* = 0.5157), demonstrating strong outcrossing dependence. Interspecific crosses in both directions ( *P. articulata* ♀ × *C. prolifera* ♂ and reciprocal) produced no fruit (Table 2), confirming effective prezygotic reproductive barriers between these coexisting species.

**TABLE 2:** Natural fruit sets of *Pholidota articulata* and *Coelogyne prolifera* in LBZ ancient tea garden over 3 years from 2019 to 2021, and the fruit sets of different hand pollination treatments in 2022 (mean ± SD).

Treatments	Species	
	*P. articulata*	*C. prolifera*
Fruit set (%) of hand‐pollination treatments in 2022 (flowers/inflorescence/plants)		
Bagging	0 (150/20/20)	0 (50/20/20)
Hand self‐pollination	0 (95/20/20)	0 (100/30/20)
Hand cross‐pollination	87.83% ± 6.94% (305/15/15) (A)	90.17% ± 10.07% (48/10/10) (A)
Interspecific pollination		
*P. articulata* (♀) × *C. prolifera* (♂)	0 (50/15/15)	—
*C. prolifera* (♀) × *P. articulata* (♂)	—	0 (50/15/15)
Natural fruit sets % (flowers/inflorescence/plants)		
2019	29.50% ± 17.80% (876/45/45) (a)	30.37% ± 24.41% (409/86/43) (a)
2020	29.79% ± 21.77% (1060/54/54) (a)	21.20% ± 18.48% (418/83/42) (b)
2021	30.71% ± 19.38% (1118/48/48) (a)	26.88% ± 23.10% (464/93/47) (ab)
Average natural fruit sets over 3 years	30.01% ± 19.86% (3054/147/147) (A)	26.23% ± 22.51% (1291/262/132) (A)

*Note:* Values in parentheses indicate sample sizes (number of flowers, number of inflorescences, number of plants). Statistically homogeneous groupings based on post hoc multiple comparison tests and one‐way ANOVA are marked by the same letter (a–b/A).

### 3.4 Pollinia Removal, Deposition, and Fruit‐Sets

**FIGURE 5:**
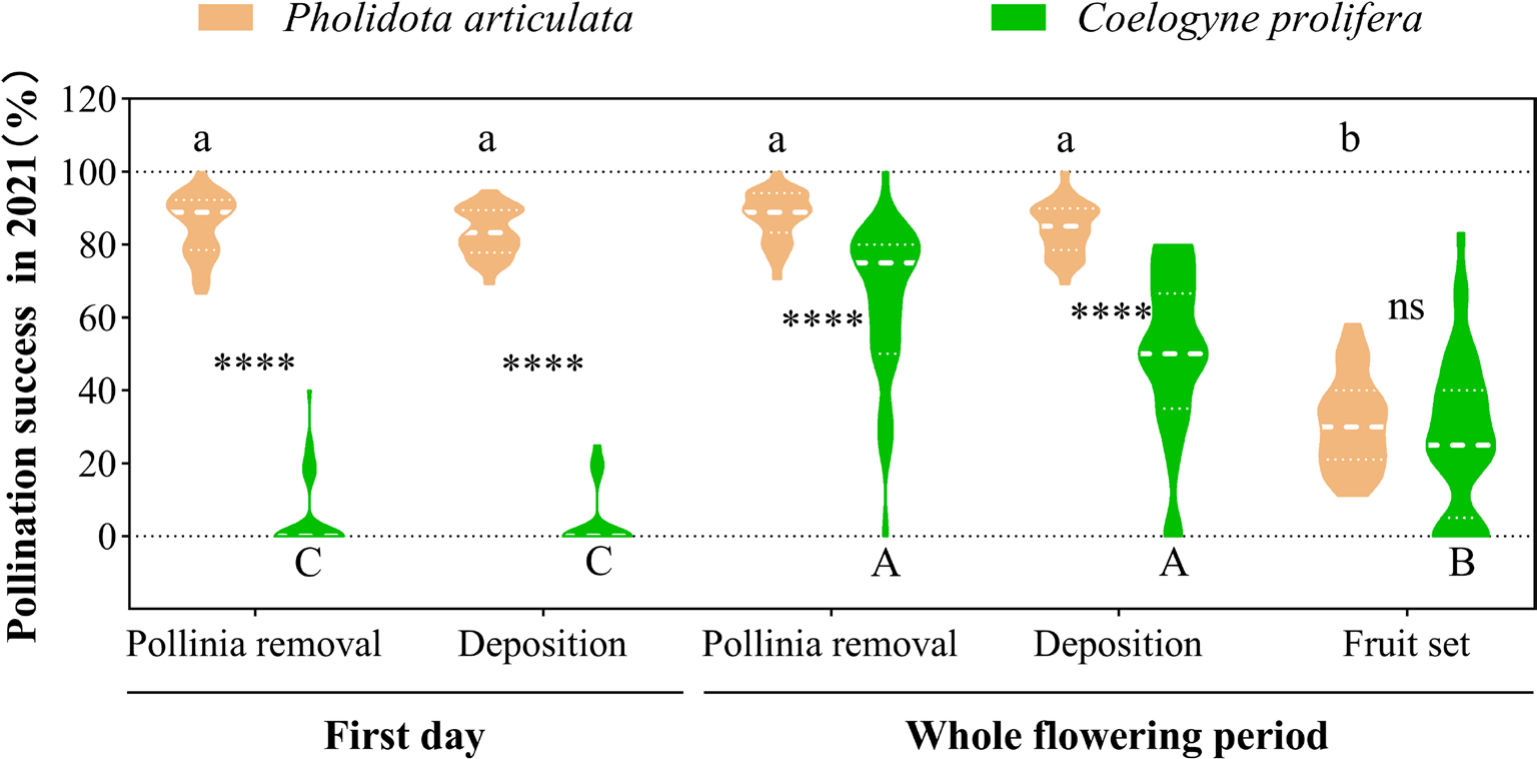
Comparative reproductive success between *P. articulata* and *C. prolifera* during first‐day versus whole flowering period. The asterisks indicate significant differences between dispersion patterns (ns, *p* > 0.05; ****, *p* < 0.0001). Statistically homogeneous groupings are marked by the same letter (a–b/A–C).

Pollination success patterns differed significantly between the two orchid species. In *P. articulata* , pollinia removal and deposition rates showed no significant differences between first‐day flowers and those during the whole flowering period (removal: *p* = 0.26; deposition: *p* = 0.54; Figure 5). However, while end‐of‐anthesis flowers achieved high pollinia deposition (84.38% ± 7.02%, *n* = 31), only 31.08% ± 12.44% (*n* = 31) developed into fruits (*p* < 0.0001; Figure 5), indicating substantial pollen discounting (63.17% of deposited pollinia). *C. prolifera* showed significantly lower pollinia removal and deposition rates in first‐day flowers compared to the whole flowering period (all *p* < 0.0001; Figure 5). Similar to *P. articulata* , flowers of *C. prolifera* throughout the flowering period had a pollinia deposition rate (50.19% ± 21.98%, *n* = 36) that was significantly higher relative to its fruit set (27.92% ± 21.21%, *n* = 36, *p* < 0.0001; Figure 5). However, *C. prolifera* exhibited lower pollen discounting (44.37%) than *P. articulata* .

Comparative analysis revealed significantly greater pollinia removal and deposition in *P. articulata* than *C. prolifera* across all floral stages (all *p* < 0.0001), yet comparable final fruit sets between species (*p* = 0.48; Figure 5). Three‐year monitoring showed stable fruit set in *P. articulata* (interannual *p* > 0.05), while *C. prolifera* exhibited significant annual variation, with 2019 fruit set exceeding 2020 (*p* < 0.05; Table 2). Despite these temporal differences, mean 3‐year fruit sets did not differ significantly between species (*p* = 0.09; Table 2).

## 4 Discussion

Our field observations in the LBZ ancient tea garden documented two coexisting, coflowering orchid species ( *P. articulata* and *C. prolifera* ) exhibiting self‐incompatibility. While historically considered relatively rare in Orchidaceae (Tremblay et al. 2005), recent studies have revealed that self‐incompatibility is in fact prevalent in several major orchid lineages, including Maxillariinae, Pleurothallidinae, and Oncidiinae (Barbosa et al. 2009; Borba et al. 2011; Castro and Singer 2019; Ricci et al. 2024). Beyond mere prevalence, research across diverse genera such as *Dendrobium*, *Pleurothallis*, and *Anathallis* further reveals the complex evolutionary interplay between self‐incompatibility and floral traits. For instance, in *Dendrobium*, self‐incompatibility is frequently correlated with nectar production despite the independent evolution of both traits (Zielińska et al. 2025), while in *Pleurothallis*, it helps maintain high genetic diversity (Borba, Semir, et al. 2001), and in *Anathallis*, different self‐incompatibility systems (gametophytic and sporophytic) are linked to taxonomic groupings (Gontijo et al. 2010). This study represents the first comprehensive investigation of pollination biology in *Pholidota* species, while expanding our understanding of *Coelogyne* pollination systems and expanding on previous reports focusing on wasp and bee pollinators (Cheng et al. 2009; Liu et al. 2013). Notably, despite the general limitation of pollinators during the rainy season, both species achieved substantially higher fruit sets (21%–31%) than the typical 2% reported for most self‐incompatible orchids (Tremblay et al. 2005), suggesting evolutionary adaptations to overcome reproductive constraints through distinct flowering strategies.

The nectar characteristics of both species reflect distinct resource allocation strategies. *P. articulata* produced significantly greater nectar volume (∼66% more) with higher sugar concentration than *C. prolifera* , despite the substantial energetic costs associated with nectar production (Southwick 1984; Koopowitz and Marchant 1998; Luyt and Johnson 2002). This difference aligns with their contrasting phenologies: *P. articulata* 's intensive 28‐day flowering period with large floral displays versus *C. prolifera* 's extended 60‐day flowering with fewer but longer‐lived flowers (13.05 ± 1.36 days vs. 4.65 ± 0.73 days). Such temporal niche differentiation may reduce interspecific competition for shared pollinators (Levin and Anderson 1970) while maintaining stable plant‐pollinator interactions over time (Zhou et al. 2016). The consistent visitation patterns of *V. velutina* and *V. mandarinia* across 3 years suggest these wasps have formed reliable mutualisms with both orchids, though visitation rates were significantly higher for *P. articulata* , likely due to its more generous nectar rewards and conspicuous floral displays.

The absence of caudicle bending movements in both species contrasts with Darwin's (1877) proposed antigeitonogamy mechanism, indicating alternative strategies have evolved. *P. articulata* achieves pollination efficiency through synchronized mass‐flowering, with 83.29% ± 6.52% of flowers pollinated on their first day, effectively minimizing geitonogamy risks despite large floral displays (e.g., Gao et al. 2012). This strategy capitalizes on pollinator attraction while concentrating reproductive events in a narrow temporal window. Conversely, *C. prolifera* 's steady‐state flowering reduces geitonogamy through spatial and temporal dispersion of floral resources, though its lower pollinator visitation rates (4.54% ± 8.60% first‐day pollination) necessitate extended floral longevity. Interestingly, although *B. breviceps* functions as a nectar robber, its frequent visits to *C. prolifera* may indirectly reduce geitonogamy by causing pollinators to encounter more empty flowers, which subsequently reduces the number of floral visits per plant and consequently decreases ineffective within‐plant pollen transfer (Irwin 2010).

Reproductive success patterns revealed fundamental differences in pollination limitation between species. While both showed substantial pollen discounting ( *P. articulata* : 63.17%; *C. prolifera* : 44.37%), only *C. prolifera* exhibited clear pollinator limitation (*p* < 0.0001 for pollinia deposition vs. fruit set). This contrast highlights the importance of evaluating both male (pollinia removal) and female (fruit set) reproductive success when assessing pollination limitation (Nilsson et al. 1992; Ackerman et al. 1997; Maad and Alexandersson 2004). The shared pollinator deposition patterns (similar head placement) create potential for reproductive interference and pollen discounting (Waser 1983; Johnson et al. 2003), though complete hybrid incompatibility prevents gene flow between these coexisting species. Our investigation of hybridization is particularly relevant in light of recent taxonomic proposals to subsume *Pholidota* under *Coelogyne* (Chase et al. 2021). The strong prezygotic barrier revealed by our reciprocal crossing experiments, however, demonstrates substantial reproductive isolation, thereby challenging the proposed synonymy.

These findings demonstrate how divergent flowering strategies can achieve comparable reproductive success in coexisting, self‐incompatible orchids. *P. articulata* 's mass‐flowering optimizes pollinator attraction and efficient pollen transfer during brief, favorable conditions, while *C. prolifera* 's steady‐state flowering ensures reproductive opportunity across variable environmental conditions. Both strategies effectively address the dual constraints of pollinator limitation and geitonogamy that typically constrain reproductive success in self‐incompatible orchids (Zimmerman and Aide 1989; Johnson and Bond 1992; Calvo 1993; Mattila and Kuitunen 2000). The maintenance of these contrasting strategies in sympatry suggests they represent evolutionarily stable solutions to similar selective pressures, with each species' strategy reflecting different optima in the trade‐off between pollinator attraction and ensuing pollination quality (Harder et al. 2004).

The stable coexistence of these orchids in the ancient tea garden ecosystem likely results from multiple factors: (1) phenological segregation of flowering peaks minimizing interspecific competition, (2) divergent resource allocation strategies between nectar production and floral longevity, and (3) a shared but flexible pollinator attraction. This system provides a compelling example of how specialized plant‐pollinator relationships can maintain species coexistence and reproductive success even in self‐incompatible species facing pollinator limitations and pollen discounting. The preservation of such intricate species interdependencies in traditional agroecosystems like ancient tea gardens highlights their conservation value as refugia for orchid diversity and specialized pollination interaction.

## Author Contributions

**Shi‐Mao Wu:** conceptualization (lead), formal analysis (lead), investigation (equal), methodology (lead), visualization (lead), writing – original draft (lead), writing – review and editing (lead). **Sheng Zhang:** investigation (equal). **Yi‐Hua Wu:** investigation (equal). **Xiang‐Gui Chen:** investigation (equal). **Jiang‐Yun Gao:** conceptualization (equal), funding acquisition (lead), writing – original draft (equal), writing – review and editing (equal).

## Funding

This work was supported by the Joint Special Project on Construction of “First‐class Universities and Disciplines” of Yunnan University (202201BF070001‐017).

## Conflicts of Interest

The authors declare no conflicts of interest.

## Data Availability

Data are available in Figshare at https://figshare.com/s/ebe37cceaef7f6be46ca.
